# Robust Frame Synchronization Scheme for Continuous-Variable Quantum Key Distribution with Simple Process

**DOI:** 10.3390/e21121146

**Published:** 2019-11-23

**Authors:** Rui Chen, Peng Huang, Dengwen Li, Yiqun Zhu, Guihua Zeng

**Affiliations:** 1 State Key Laboratory of Advanced Optical Communication Systems and Networks, Center for Quantum Sensing and Information Processing, Shanghai Jiaotong University, Shanghai 200240, China; 118034910040@sjtu.edu.cn (R.C.); dengwen_li@sjtu.edu.cn (D.L.); ghzeng@sjtu.edu.cn (G.Z.); 2 School of Electronic Information, Shanghai Dianji University, Shanghai 201306, China; zhuyiq@sdju.edu.cn

**Keywords:** continuous-variable quantum key distribution, frame synchronization, phase shifts

## Abstract

In continuous-variable quantum key distribution (CVQKD) systems, high-quality data synchronization between two legitimate parties, Alice and Bob, is the premise of the generation of shared secret keys. Synchronization with specially designed frames is an efficient way, but it requires special modulating devices to generate these special frames. Moreover, the extra requirement of special modulating devices makes it technically impossible for some passive preparation schemes. We propose a novel approach to realize synchronization in this paper, which is different from those special-frame-based methods. In our proposed scheme, Alice publishes parts of the original signals as the synchronization frames and Bob takes these frames to perform the synchronization algorithm. Besides, a synchronization feature is applied to deal with phase shifts. The simulation results based on practical data demonstrate that the proposed synchronization scheme not only maintains a high success rate but simplifies the data processing flow at the same time, which dramatically reduces the computational complexity.

## 1. Introduction

Quantum key distribution (QKD) has become a popular topic for its confidentiality, which allows two legitimate parties far away to share secure secret keys through an untrusted channel with unconditional security [1,2,3]. Generally speaking, dominated protocols of QKD can be divided into two categories, which can be defined as discrete-variable QKD (DVQKD) [4,5] and continuous-variable QKD (CVQKD) [2,6,7]. In the DVQKD scheme, secret keys would be encoded on polarization states, phases, or other discrete variables of single photons. In the CVQKD system, information is encoded on the position and momentum quadrature of the light field. Then the receiver, Bob, uses homodyne detectors or heterodyne detectors to measure one or both quadrature components. By controlling excess noise, the CVQKD system can be achieved beyond 100 km at present through standard single-mode optical fibers [8,9]. Moreover, the CVQKD can utilize existing optical communication components, which provides a prospect of good integration with classical optical communications.

In a typical CVQKD system, Alice first prepares quantum states. Then secret information, which is produced from the true random number generator, is encoded on the position or momentum quadrature of quantum states by amplitude and phase modulations. After that, the modulated quantum states, which can be expressed as $|x_{A}+ip_{A}\rangle$, are sent to Bob through a quantum channel. Affected by quantum noise and other classical noise, Bob will receive a noise state $|x_{B}+ip_{B}\rangle$. Transmission of the quantum signal over a lossy and noisy channel may highly affect the performance of the frame synchronization algorithm. For homodyne detection, Bob randomly chooses *X* or *P* measurement bases. Afterward, he compares them with Alice’s bases and selects the variables with the same bases. After reconciliation and privacy amplification processes, Alice and Bob will share the same key data.

It is worth noting that synchronization in CVQKD plays an important role. Simply speaking, if the data of Alice and Bob are not aligned, decoding key information in Bob’s side will be independent with the one Alice prepared, which results in inconsistent secret key strings after the reconciliation process, and thus deteriorates the overall performance. In a CVQKD system, clock synchronization makes two communication entities share the same clock to acquire accurate data. So far, clock synchronization schemes include the transmitted local oscillator (TLO) [10] and local local oscillator (LLO) schemes [11,12], where the latter can thoroughly remove the related loopholes [13,14] introduced by transmitted LO signals. Frame synchronization determines the head of every signal string, so even minor synchronization errors will lead to a huge decrease in the mutual information between Alice and Bob. Most previous methods tend to use specific modulations to generate synchronization frames, and the well-organized frames are periodically inserted into the data frames by Alice [15,16,17]. Although these methods have been proved efficient in some situations, the performances of them are far from satisfied under low signal-to-noise ratio (SNR) scenarios. To overcome this shortcoming, a frame synchronization scheme based on phase disassembling and matching by comparing correlation was put forward [18]. However, in the synchronization procedure, computing correlation requires a lot of multiplication and the previous calculations cannot be reused in subsequent calculations. An expected frame synchronization scheme should have high-efficiency at a low SNR and low computational complexity at the same time.

Besides, in practical CVQKD applications, the quantum state will suffer unpredictable nonlinear effects, and the quadrature components of the optical field of quantum states will suffer phase shifts during signal transmissions [19,20], which means a well-designed frame synchronization scheme should be well tolerable of phase shifts. If the two legitimate entities have been successfully synchronized, the phase shifts can be removed by phase compensation methods [21,22]. So the synchronization process is usually previous to phase compensation, and frame synchronization should tolerate a certain amount of phase shifts.

To simplify the frame synchronization scheme and improve the efficiency and robustness of the CVQKD system, we propose a novel scheme here. In particular, a new feature is designed, which can tolerate phase shifts and synchronize in a strong noise environment. Each synchronization process requires only a few addition and subtraction operations and a Hamming distance comparison. In particular, we analyze the performance of this method under different phase shifts and various SNR settings. The results show that this scheme can tolerate different phase shifts and performs well at a low SNR. Moreover, the proposed scheme also keeps a good balance between performance and computational complexity.

The rest of the paper is organized as follows: In Section 2, we first introduce the synchronization process and the designed feature in detail, then illustrate the reason why this feature can tolerate different phase shifts. In Section 3, the simulations of the proposed algorithm under different parameter settings are performed. Finally, a brief conclusion is given in Section 4.

## 2. Synchronization in CVQKD

In the common frame synchronization scheme of CVQKD [15,16], the training frames should be added to realize data synchronization between Alice and Bob. The synchronization frames are modulated into a special format, known by Alice and Bob and can be easily recognized. However, in some special CVQKD schemes, it is difficult or even impossible to add synchronization frames into key data by modulation devices, such as the passive-state-preparation CVQKD scheme [23]. In the passive-state-preparation CVQKD scheme, Alice can split the output of a thermal source by a beam splitter and one mode is measured by herself while the other mode is transmitted into the other legitimate entity, Bob. As Alice directly split the output of the source and did not use any modulation devices to encode information onto the mode, it is hard to add synchronization frames into the signal. This inspired us to look for ways to synchronize using random number strings. Moreover, the traditional synchronization process usually needs a high range switch of light intensity. These light switching schemes make CVQKD systems more complicated and unstable.

These issues prompted us to improve the training-frame-based scheme into a modulation-free one without specified synchronization frames. In addition, phase drifts between the LO and signal will introduce extra trouble into the synchronization process. A practical scheme should overcome the phase drifts to successfully implement synchronization. In classical optical and wireless communications, synchronization can be performed by measuring the Hamming distance between the outputs of the transmitter and the received signals [24]. The Hamming distance equals to the different bits of two 0–1 sequences $S_{1}$, $S_{2}$. Comparing to the calculation of correlation, the Hamming distance has low computation complexity. Here, we can first convert the signals into 0-1 sequences by certain algorithms and then measure their Hamming distance. It should be mentioned that these transform algorithms must be robust against different environment noises.

### 2.1. Finding Robust Feature

In this part, we will mainly analyze the influence of the phase shift on the synchronization process, then introduce a robust feature. Alice sends quantum states $|X_{A}+iP_{A}\rangle$ to Bob through a quantum channel with Gaussian distributed noise ξ and phase shift Δφ. In fact, the noise in the channel can be divided into two parts. The one added by the channel is called channel-added noise. It can be expressed as $\chi_{line}=1/T-1+\varepsilon_{c}$ (*T* is the transmittance of the quantum channel and $\varepsilon_{c}$ means the excess noise). The other noise is added by the thermal motion of detectors, called detection-added noise. The detection-added noise can be expressed as $\chi_{hom}=\left(1-\eta +\nu_{el}\right)/\eta$ (homodyne detector) or $\chi_{het}=\left(1+\left(1-\eta \right)+2\nu_{el}\right)/\eta$ (heterodyne detector), in which η means the attenuation factor and $\nu_{el}$ means the thermal noise caused by electronics in homodyne detectors or heterodyne detectors. And the total noise referred to the channel input can be given by $\chi_{tot}=\chi_{line}+\chi_{hom}/T$. From reference [25], $X_{A}$ and $P_{A}$ are Gaussian distributed random variables. For simplicity, here we temporarily omit the attenuation. When Bob measures the quantum states $|X_{B}+iP_{B}\rangle$ with a homodyne or a heterodyne detector, the measurement results can be expressed as

(1) $$X_{B}=A\cos\left(\theta +\Delta \varphi \right)+\xi =X_{A}\cos\left(\Delta \varphi \right)-P_{A}\sin\left(\Delta \varphi \right)+\xi ,$$

(2) $$P_{B}=A\sin\left(\theta +\Delta \varphi \right)+\xi =P_{A}\cos\left(\Delta \varphi \right)+X_{A}\sin\left(\Delta \varphi \right)+\xi ,$$

where $X_{A}=A\cos\left(\theta \right),P_{A}=A\sin\left(\theta \right)$. Without loss of generality, in the following analyses, we assume that ξ is a Gaussian distributed random variable with expectation 0 and variance σ, and the phase shift δφ keeps the same within a small period time. From the above formula, we know that if we want to eliminate the effect of phase shifts in synchronization, some stable features must be found.

To cope with the phase shifts, here we introduce a new operator $\Delta X_{(AorB),n}=\sum_{i=1}^{L}X_{(AorB),n+i}-\sum_{i=1}^{L}X_{\left(AorB,n-i\right)}$ ($\Delta P_{(AorB),n}$ can be defined in the same way) called incremental label. Now we investigate the effect of phase drift on it. The conditional expectation of the operator can be written as

(3) $$\begin{array}{cc} E(\Delta X_{\left(B,n\right)}|\Delta X_{\left(A,n\right)}=V_{th}) & =E\left[\Delta X_{\left(A,n\right)}\cos\Delta \varphi -\Delta P_{\left(A,n\right)}\sin\Delta \varphi |\Delta X_{\left(A,n\right)}=V_{th}\right]\\ \; & =V\cos\Delta \varphi , \end{array}$$

where $V_{th}$ is a positive threshold and Δφ∈(-π,π) means phase shift. If Δφ∈(-π/2,π/2), then the conditional expectation is positive. Otherwise, it will be a negative one. sign(x) is the sign function that outputs the sign of number *x*. The sign of the above conditional expectation is,

(4) $$\begin{array}{c} sign\left[E(\Delta X_{\left(B,n\right)}|\Delta X_{\left(A,n\right)}=V_{th})\right]=sign\left(V\cos\Delta \varphi \right)=sign\left(\cos\Delta \varphi \right). \end{array}$$

So when $\Delta X_{\left(A,n\right)}$ is larger than a significant positive threshold, the operator $\Delta X_{\left(B,n\right)}$ can be regarded as a quasi-stable feature. Here we can apply this operator on a string of random numbers and yield a binary sequence. We first apply it on Alice’s key string to get the binary sequence $S_{A}$, and then apply it on Bob’s one to get $S_{B}$. Suppose that the phase shift Δφ keeps the same for a while; if cosΔφ is positive, the result will be $S_{A}=S_{B}$, else $S_{A}=-S_{B}$.

In the above discussion, we do not consider one case that Δφ is approaching ±π/2. In fact, when Δφ is close to ±π/2, $X_{B}$ is similar to the quadrature component $P_{A}$. When this happens, another conditional expectation should be explored,

(5) $$\begin{array}{c} sign\left[E(\Delta X_{\left(B,n\right)}|\Delta P_{\left(A,n\right)}=V_{th})\right]=sign\left(-V\sin\Delta \varphi \right)=-sign\left(\sin\Delta \varphi \right). \end{array}$$

From the above expression, we can see that if cosΔφ approximately becomes 0, taking component *P* into consideration is another good way. In the following section, we will show how the above conclusion can be applied to real synchronization.

The conditional expectation of the incremental label and its sign give us some ideas that the sign of $\Delta X_{(AorB),n}$ is stable in a noisy environment, and a robust 0–1 string can be constructed in this way. The relationship between the conditional expectation and phase shifts can help to deal with the phase drift problems in the synchronization process. This will be elaborated on in the following section.

### 2.2. Incremental Label

**Figure 1:**
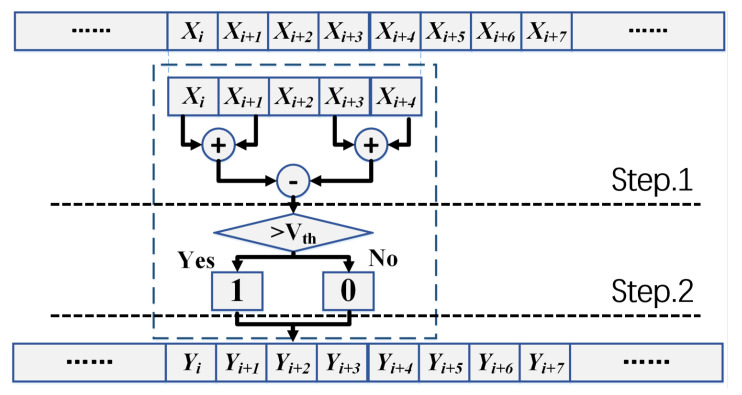
The proposed synchronization scheme.

Based on the above analysis, the incremental label can be constructed. This labeling method will transform a random number sequence *X*, which can be expressed as $\left(x_{1},x_{2},\cdots \right)$ into a binary sequence *Y*$\left(y_{1},y_{2},\cdots \right)$ by the rules:

Step 1. Sum the next *L* numbers of the current position, such as shown in Figure 1 $X_{i+3},X_{i+4}$ for current position $X_{i+2}$ and L=2. Then subtracting the sum of the former *L* numbers, the output is used as a descriptor. We call the 2L+1 interval a transformation unit.

Step 2. If $\sum_{j=i+1}^{i+L}x_{j}-\sum_{j=i-L}^{i-1}x_{j}>V_{th}$, we mark this position with symbol “1” ($y_{i}=1$). If $\sum_{j=i+1}^{i+L}x_{j}-\sum_{j=i-L}^{i-1}x_{j}<=V_{th}$, we mark this position with symbol “0” ($y_{i}=0$).

Step 3. After all the received signals are marked, the synchronization process begins. Every successive *N* bits of conversion sequence *Y* are seen as a feature, and we can calculate the Hamming distance of the two signal sequences to measure their similarity.

It should be mentioned that noise with zero expectation will be suppressed and their impact on synchronization is weakened. This transformation method is simple and efficient, and we will show its performance in the next section and analyze computation complexity in the computational analysis section.

To compete with phase drifts, the sender Alice can prepare four transformation sequences of her synchronization frames. Firstly, Alice generates the binary sequences $TX_{A}$ and $TP_{A}$ by using the rules listed in steps 1 and 2. Then their complements, $\bar{TX_{A}}$ and $\bar{TP_{A}}$, can directly get a not operator. For example, if $TX_{A}$ is “0101,” then $\bar{TX_{A}}$ is “1010;” the rules are the same for $TP_{A}$ and $\bar{TP_{A}}$. Bob also transforms the received $X_{B}$ or $P_{B}$ with the same rules.

After the sequence transformations, the similarity can be measured by calculating the Hamming distance between the transformed sequences of Alice’s synchronization symbols and every segment of Bob’s received signal. Here we want to make the cost function reach its peak value when synchronization succeeds, so the cost is rewritten

(6) $$D\left(TX_{A},TX_{B}\right)=n-H\left(TX_{A},TX_{B}\right),$$

where $D(X_{1},X_{2})$ means the similarity of sequences $X_{1}$ and $X_{2}$, and $H(X_{1},X_{2})$ means the Hamming distance of $X_{1}$ and $X_{2}$. Here, we define a new function,

(7) $$F\left(A,B\right)=max\left[D\left(TX_{A},TX_{B}\right),D\left(\bar{TX_{A}},TX_{B}\right),D\left(TP_{A},TX_{B}\right),D\left(\bar{TP_{A}},TX_{B}\right)\right].$$

The location of synchronization is where the function F(A,B) reaches its peak value.

### 2.3. The Synchronization Flow

From the above derivations, we have now found a stable feature to endure phase drifts. The following synchronization scheme is based on this feature.

**Figure 2:**
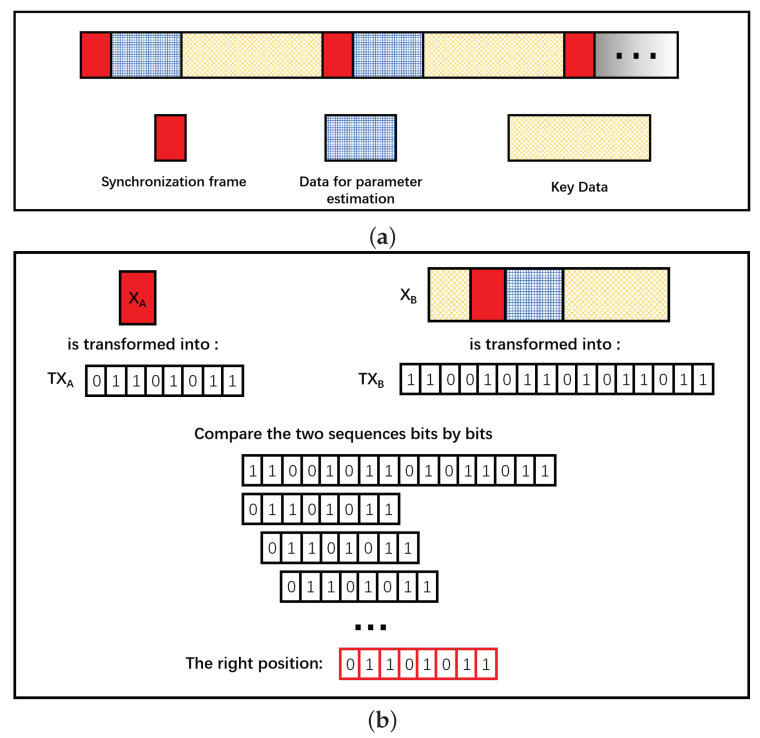
(**a**) The structure of the data frame in the continuous-variable quantum key distribution (CVQKD) system; (**b**) the synchronization process in the CVQKD system.

Step 1. Alice (the sender) selects parts of the random strings as the synchronization frame (see Figure 2a).

Step 2. Alice transforms the selected sequences into 0–1 sequences using the incremental label algorithm proposed above. Both *X* and *P* components must be transformed. We the get two 0–1 sequences: $TX_{A}$ and $TP_{A}$.

Step 3. Alice publishes the two 0–1 sequences $TX_{A}$ and $TP_{A}$ through the classical channel.

Step 4. Bob transforms $X_{B}$ or $P_{B}$ into 0–1 sequences by the incremental label algorithm, and matches them to the received two 0–1 sequences $TX_{A}$ and $TP_{A}$ bits by bits. Then he calculates the function $F\left(A,B\right)=max\left[D\left(TX_{A},TX_{B}\right),D\left(\bar{TX_{A}},TX_{B}\right),D\left(TP_{A},TX_{B}\right),D\left(\bar{TP_{A}},TX_{B}\right)\right]$ (see Figure 2b).

Step 5. Alice and Bob synchronize at the position where the function F(A,B) reaches its peak value.

**Figure 3:**
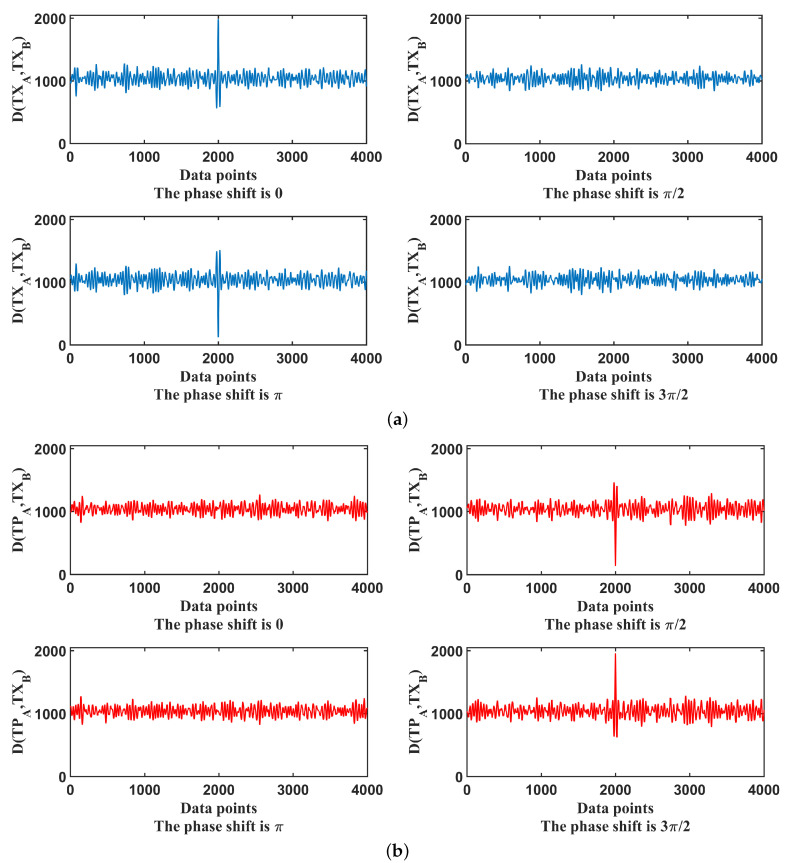
(**a**) Cost function $D(TX_{A},TX_{B})$ under different phase shifts; (**b**) Cost function $D(TP_{A},TX_{B})$ under different phase shifts.

To verify the correctness of the proposed scheme, the synchronization process is simulated as follows. The encoded random strings in Alice’s side are $X_{A}$ and $P_{A}$, and Bob’s received signals are $X_{B}$ and $P_{B}$. Figure 3 shows the cost function $D(TX_{A},TX_{B})$ and $D(TP_{A},TX_{B})$ under different phase shifts.

The cost function $D(TX_{A},TX_{B})$ reaches its peak value when the synchronization succeeds if the phase shift is 0. However, the value will bottom out when the phase shift comes to π. There will be no peak or valley values when the phase shift reaches π/2 or 3π/2. Similarly, the cost function $D(TP_{A},TX_{B})$ has a valley value corresponding to the π/2 phase shift situation while it reaches its maximum if the phase shift changes to 3π/2. The results also provide further evidence on how reasonable and feasible the proposed new function is in Equation (7).

## 3. Performance Analysis

To explore the influence of SNR (signal-to-noise ratio) and phase shifts on the performance of the proposed frame synchronization algorithm, we prepare several strings of data with natural Gaussian distributions generated from ASE output signals with length 200,000. We add Gaussian white noise of different variance to the output signal to simulate different noise environments. Here we randomly select some segments of the signals as synchronization frames, and we define the proportion of the times of successful synchronization as the success rate. To improve the success rate, the parameter *L* should be longer than 10 and the threshold $V_{th}$ could be set as the variance of the received signals.

### 3.1. Performance Influenced by Phase Shifts

**Figure 4:**
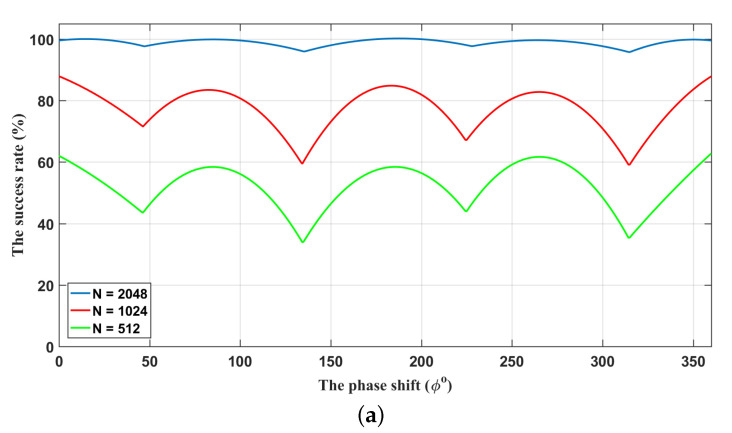

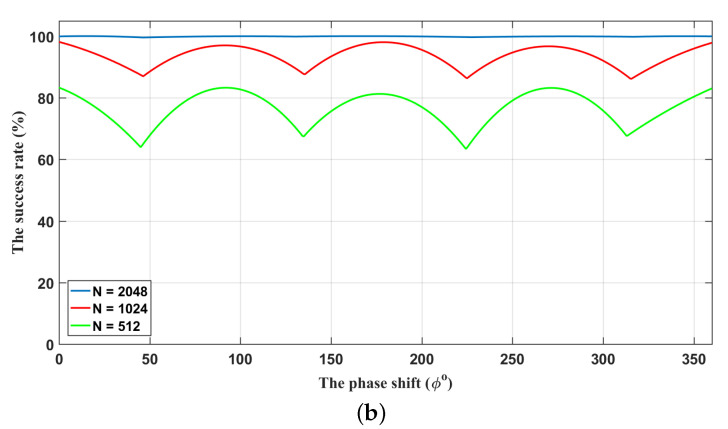
The relationship between the phase shifts and the success rate of synchronization. (**a**) Synchronization by using only one quadrature component; (**b**) Synchronization by using two quadrature components. The lengths of synchronization frames from bottom to top are N=512,1024,2048. The SNR is −13 dB.

Figure 4a,b shows that the influence of different phase shifts on the proposed algorithm. The synchronization process operates at an SNR of −13 dB with feature-lengths of N=512,1024,2048. It can be found that increasing the feature-length can significantly improve performance. We can find the success rate will bottom out for the phase shifts $\Delta \varphi =45^{\circ },135^{\circ },225^{\circ },315^{\circ }$, and the success rate seems to be unsatisfactory. This is because when phase shifts take these values, cos(Δφ)=sin(Δφ), the proposed algorithm merely deals with one quadrature *X* or *P*. If Bob applies a heterodyne detector to measure $X_{B}$ and $P_{B}$ simultaneously, these two values can both be used to perform synchronization and, thus, better results can be achieved. The above analyses show that although the proposed scheme is more suitable for protocols based on heterodyne detection, it can also be applied to homodyne-based protocols. Using a heterodyne detector to measure $X_{B}$ and $P_{B}$ simultaneously in the final matching step will get a better result. Merely considering one quadrature *X* or *P* can also synchronize well when the phase shifts occur.

### 3.2. Synchronization with Different SNRs

**Figure 5:**
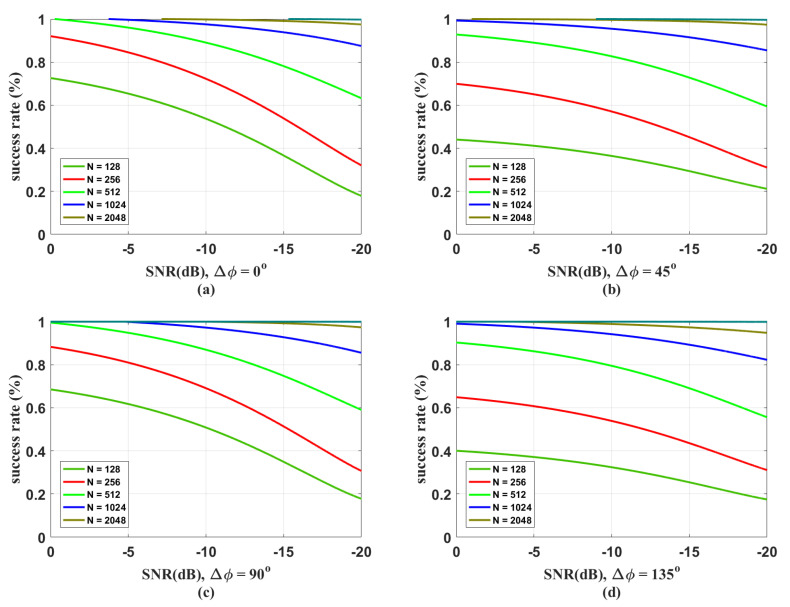
The success rates of the proposed algorithm under different SNR conditions. (**a**) Phase shift $0^{\circ }$; (**b**) phase shift $4^{\circ }$; (**c**) phase shift $90^{\circ }$; (**d**) phase shift $135^{\circ }$. The lengths of synchronization frames for the curves from bottom to top are N=128,256,512,1024,2048.

In Figure 5a–d, we explore the performance of the proposed synchronization algorithm under different SNR conditions with phase shifts $\Delta \varphi =0^{\circ },45^{\circ },90^{\circ },135^{\circ }$, respectively. We find that the decrease in success rate caused by the low SNR can be effectively improved by increasing the length of features. From the above discussions, we know that the phase shifts $\Delta \varphi =45^{\circ },135^{\circ }$ are two points that the success rate reaches its minimum value, which is also demonstrated in these figures. When setting the feature-length as N=2048, despite the phase shifts, the success rate will be higher than 90% when SNR is larger than −20 dB. Usually, the data block of a CVQKD system with a repetition rate of 100 MHz has 100,000 characters. If we set the feature-length *N* to maximal 2048, the fraction, which is used for synchronization, is 2.048%. Our synchronization scheme requires only a small sacrifice of data.

### 3.3. Algorithm Complexity

Complexity is another important factor for a practical synchronization algorithm. In a practical CVQKD system, the synchronization algorithm should work in real-time for high efficiency, otherwise, it will require a mass of storage to store all the received data. At first sight, according to the algorithm flow, every step has 2NL times added to operations (the transformation unit length *L* is mentioned above, usually it can be set as L=13), *N* times of subtraction operation and an N-bit Hamming distance operation. This is because data reuse is not considered. In a practical synchronization, every synchronization step needs only 2L times add operations except for the first step, 1 time subtraction operation, and an N-bit Hamming distance operation. The previously stored transform results can be used. It should be noted that the expression $\sum_{j=i+1}^{i+L}x_{j}-\sum_{j=i-L}^{i-1}x_{j}<=V_{th}$ (center on $X_{i}$) just needs to be calculated one time for every step and a unique binary mark will be allocated to the corresponding location $Y_{i}$. There is no need to calculate it again when generating the next feature. The analysis shows that the proposed scheme can save computation resources and maintain good performance.

Comparing to the frame synchronization method based on the correlation calculation [18], the proposed algorithm has a much lower computation complexity. In particular, if the feature-length is *N*, the calculation of a correlation needs multiplication operations to get the result of every $x_{i}y_{i}$ and N-1 times add operations for the final result. Furthermore, the times of added to operations can be well reduced. One can first divide the entire sequence into several pairs, then calculate the sums of every pair to get the first N/2 results. After the final iterative process, the add operation times can be reduced to $log_{2}N$. However, there are not any multiplication operations in the proposed scheme, which significantly reduces the computational complexity (see Table 1).

### 3.4. Security and Adaptivity Analysis

The realistic system may incur loopholes due to the imperfections of the implementation process, although the CVQKD protocols are theoretically proven to be secure. Traditional frame synchronization methods are performed by alternately transmitting a strong pulse. Although there has not been any practical attack on these frame synchronization schemes, the use of strong pulses can be manipulated, which may incur potential loopholes. Moreover, the frame synchronization methods based on the designing of the special frame may also introduce potential risks, since the synchronization frames can be distinguished from the key data. A well-designed synchronization method should conceal its synchronization frames into key data so that it is hard for an eavesdropper to distinguish them.

Synchronization frames of this proposed scheme are similar to data frames but they are uncorrelated, which is different from the traditional schemes. In our proposed synchronization scheme, we regard parts of the signals as synchronization frames, so the signals and synchronization frames have the same distribution and the same power. If an eavesdropper intends to attack the CVQKD system through the potential loopholes in the frame synchronization method, she must distinguish the synchronization frames from the quantum signals. So she will detect the quantum signals and this will inevitably cause an increase in excess noise. Her attacks will be then found by the legitimate parties in the following key generation steps. Although the synchronization method in this article uses parts of data as the synchronization frame. The revealing of these synchronization frames does not leave any useful information about the secret key.

In our synchronization scheme, a fraction of data is used as a reference frame. It means that the scheme will also work at the cost of a slight drop in the secret key rate as the previously proposed frame synchronization schemes. We can evaluate the influence of using synchronization frames on the secret key rate when considering the finite-size effects,

(8) $$K_{finite}=\frac{n}{N}\left(\beta I_{AB}-\chi_{BE}-\Delta \left(n\right)\right),$$

where $I_{AB}$ means the mutual information between Alice and Bob; $\chi_{BE}$ is the Holevo bound on the information between Bob and Eve; Δ(n) can be approximated to $7\sqrt{\frac{log_{2}\left(2/\bar{\varepsilon }\right)}{n}}$; *N* denotes the block length and *n* denotes the size of the samples used for final key generation.

**Figure 6:**
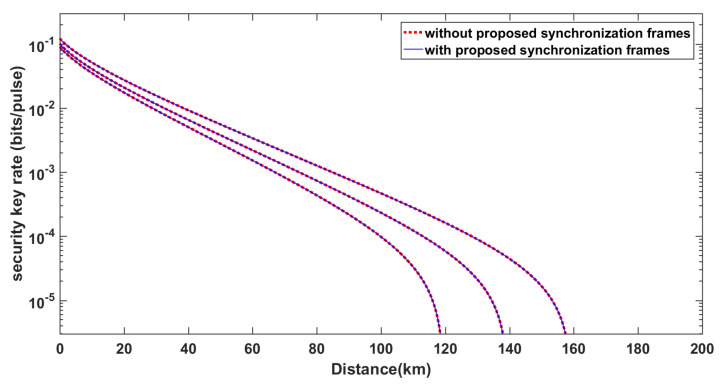
The secret key rates with or without the considerations of using the proposed frame synchronization. The curves from left to right respectively correspond to block lengths of $N=10^{10},10^{11},10^{12}$, respectively. The solid blue lines correspond to the secret key rates without considering the cost of synchronization. The dotted red lines correspond to the secret key rates with consideration of using the proposed frame synchronization.

Figure 6 shows the secret key rate curves with or without our synchronization scheme. In the simulation, the lengths of synchronization frames are all $2^{12}$ in different scenarios; the reconciliation efficiency β is set to β=0.956; the attenuation coefficient of optical fiber is set to γ=0.2 dB/km and the excess noise of the quantum channel is $\epsilon_{e}=0.01$, as experimentally shown in Ref [8]. These two types of curves almost overlap, which indicates that the data sacrificed for synchronization have no significant influence on the secret key rate.

**Figure 7:**
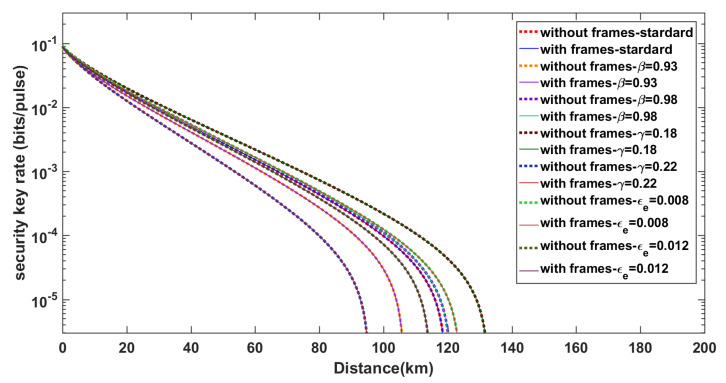
Comparison of how the agreement of the two curves (the secret key rate curves with or without our synchronization scheme) changes under different conditions.

In Figure 6, we show the two types of curves (the secret key rate curves with or without our synchronization scheme) are almost consistent. Whether this consistency changes as the parameters β, γ, and $\epsilon_{e}$ change is worth exploring. Figure 7 reveals that the performance of our algorithm does not deviate under different parameter-settings. We keep the lengths of synchronization frames equal to $2^{12}$. The standard setting in Figure 7 is the block length $N=10^{10}$, β=0.956, γ=0.2 dB/km, and $\epsilon_{e}=0.01$. Keeping other parameters the same, we separately set the parameters β=0.93,0.956,0.98, γ=0.18,0.2,0.22 dB/km and $\epsilon_{e}=0.008,0.01,0.012$. It can be seen that the corresponding curves are all nearly coincident. Essentially, it is because the synchronization scheme uses just a little data for the synchronization frames.

Another important thing needs to be considered here is whether the proposed frame synchronization scheme is valid or not when the attenuation of the quantum channel fluctuates. Actually, except the threshold $V_{th}$ (the threshold $V_{th}$ could be the variance of the received signal) in the labeling procedure must be changed with the value of the received signal, the whole algorithm flow is independent of fluctuations of channel attenuation. The algorithm generates incremental labels by considering relative values rather than absolute values. So the algorithm can resist attenuation fluctuation to some extent.

**Figure 8:**
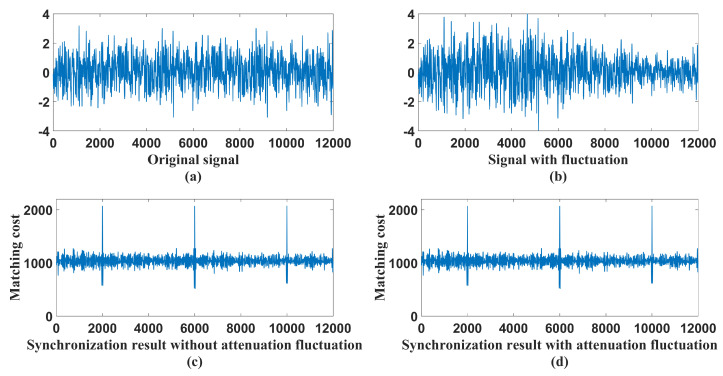
The influence of attenuation fluctuation on the proposed algorithm. (**a**) Signal without attenuation fluctuation; (**b**) Signal with attenuation fluctuation; (**c**) Synchronization result of the signal without attenuation fluctuation; (**d**) Synchronization result of signal with attenuation fluctuation.

We simulate the process of quantum channel attenuation fluctuation and test the performance of the proposed synchronization algorithm in this condition. We first simulate the synchronization performance of Bob receiving signals through a constant attenuation channel. Afterward, we change the channel into a fluctuation one and compare the matching cost of these two situations (see Figure 8). The matching cost curve changes little despite the existence of channel attenuation (Figure 8b,d are almost the same). Therefore, attenuation fluctuation has a limited effect on synchronization.

## 4. Conclusions

Synchronization is a crucial step in the CVQKD. Traditional methods always need to construct special synchronization frames. We propose here a simple and robust synchronization scheme without particularly designing the frame for the CVQKD system. In the proposed scheme, the sender Alice only needs to transmit parts of the quantum signals as synchronization frames to the receiver Bob. A novel feature is designed to help find the correct synchronization location. The analysis of our scheme shows that the feature we designed can tolerate phase shifts among range (0,2π) and the scheme can synchronize well under low SNR conditions. The simulations of the scheme under different parameter settings indicate that the performance can be significantly improved with increasing feature-length. Moreover, the proposed feature has lower computational complexity while maintaining a good synchronization performance.

## Figures and Tables

**Table 1:** Comparison of the algorithm complexity.

Items	Add/Substract	Multiplication	Comparation	Hamming Distance
Correlation	$log_{2}N$	*N*	0	0
Incremental labeling	2L+1(L<<N)	0	1	*N*
